# BMP-2 signaling in ovarian cancer and its association with poor prognosis

**DOI:** 10.1186/1757-2215-2-4

**Published:** 2009-04-14

**Authors:** Cécile Le Page, Marie-Line Puiffe, Liliane Meunier, Magdalena Zietarska, Manon de Ladurantaye, Patricia N Tonin, Diane Provencher, Anne-Marie Mes-Masson

**Affiliations:** 1 Centre de recherche du Centre Hospitalier de l'Université de Montréal (CR/CHUM)/Institut du cancer de Montréal, Montréal, Canada; 2 Departments of Human Genetics and Medicine, McGill University, Canada; 3 The Research Institute of the McGill University Health Centre, Montréal, Canada; 4 Départment de gynécologie et obstétrique, Université de Montréal, Montreal, QC, Canada; 5 Départment de Médicine, Université de Montréal, Montréal, Montréal, Canada

## Abstract

**Background:**

We previously observed the over-expression of BMP-2 in primary cultures of epithelial ovarian cancer (EOC) cells as compared to normal epithelial cells based on Affymetrix microarray profiling [1]. Here we investigate the effect of BMP-2 on several parameters of ovarian cancer tumorigenesis using the TOV-2223, TOV-1946 and TOV-112D EOC cell lines.

**Methods:**

We treated each EOC cell line with recombinant BMP-2 and assayed various parameters associated with tumorigenesis. More specifically, cell signaling events induced by BMP-2 treatment were investigated by western-blot using anti-phosphospecific antibodies. Induction of *Id1, Snail *and *Smad6 *mRNA expression was investigated by real time RT-PCR. The ability of cells to migrate was tested using the scratch assay. Cell-cell adhesion was analyzed by the ability of cells to form spheroids. We also investigated BMP-2 expression in tissue samples from a series of EOC patients.

**Results:**

Treatment of these cell lines with recombinant BMP-2 induced a rapid phosphorylation of Smad1/5/8 and Erk MAPKs. Increased expression of *Id1*, *Smad6 *and *Snail *mRNAs was also observed. Only in the TOV-2223 cell line were these signaling events accompanied by an alteration in cell proliferation. We also observed that BMP-2 efficiently increased the motility of all three cell lines. In contrast, BMP-2 treatment decreased the ability of TOV-1946 and TOV-112D cell lines to form spheroids indicating an inhibition of cell-cell adhesion. The expression of BMP-2 in tumor tissues from patients was inversely correlated with survival.

**Conclusion:**

These results suggest that EOC cell secretion of BMP-2 in the tumor environment contributes to a modification of tumor cell behavior through a change in motility and adherence. We also show that BMP-2 expression in tumor tissues is associated with a poorer prognosis for ovarian cancer patients.

## Background

Epithelial ovarian cancer (EOC) is the second most common gynecological cancer and accounts for nearly half of all deaths associated with gynecological pelvic malignancies. Largely asymptomatic, over 70% of patients diagnosed with ovarian cancer at an advanced stage of the disease. Early detection is rare and screening programs in the general population have been unsuccessful. Recent studies have analyzed gene expression patterns to identify the molecular events involved in the development of cancer and to uncover diagnostic and prognostic markers. This approach, applied to ovarian cancer [2-10], has resulted in the identification of several hundred genes differentially expressed between NOSE (normal ovarian surface epithelia) and EOC [11]. In a previous study from our group [1] several candidate genes that discriminate NOSE from EOC cells were identified and validated by real time RT-PCR. The differential expression of one of these candidates, bone morphogenic protein-2 (BMP-2), was further validated by immunohistochemistry (IHC) of patient tissue samples [1].

The biological role of BMP-2 in ovarian cancer has not been elucidated. BMPs are members of the TGF-β superfamily, which play an important role in embryonic development events, such as gastrulation, neurogenesis, hematopoiesis and apoptosis [12,13]. Recent studies have suggested that some BMPs are implicated in cancer development [14] as shown in breast and prostate cancer (reviewed in [15,16]). The effects of BMP-2 on cancer cells are controversial and are perhaps dependent on the tissue and environment where they are expressed [17]. For example, BMP-2 has been shown to stimulate the growth of pancreatic carcinoma cells and prostate cancer cells in absence of androgen [18,19]. On the other hand, BMP-2 clearly inhibits the growth of tumor cells of many origins including cancers arising from thyroid, androgen-dependent prostate in presence of androgen, myeloma, gastric and pancreatic cells [14,18-22]. In cancer cells, BMP-2 was found to suppress apoptosis induced by TNFα or by serum deprivation [23-25]. In ovarian cancer, overexpression of BMP-2, BMP-4 and BMP-7 mRNAs have been reported as dysregulated by microarray analyses [1,7,8]. A recent study has demonstrated the involvement of BMP-4 in the epithelial mesenchymal transition in human ovarian cancer cells [26]. Since BMP-2, along with family members BMP-4 and BMP-7, share the same receptors they may have similar effects. However, the binding affinity of BMPs on these receptors and subsequent receptor oligomerization are different which may lead to different downstream signaling and biological effects in response to BMPs [15,27].

BMP-2 acts via two types of serine/threonine receptors [27]. Type I receptors are BMPR1a/Alk3 and BMPR1b/Alk6 and type II receptors are BMPR2 and ActRIIA. Type I receptors are phosphorylated by type II receptors after oligomerization occurs. Of the two signaling pathways for BMP, the Smad-dependent pathway appears to be the most important. Smad 1/5/8 are mediators of BMPRIa and BMPRIb whereas Smad6 and Smad7 are the inhibitory Smads of this pathway [28] Phosphorylated Smad 1/5/8 forms a complex with Smad4 and translocate in the nucleus (review [15]). The Smad-independent pathway activates TAK1, which can lead to MAPK activation as well as Akt and NF-kappaB activation [29,30]. The most characterized target genes of the BMP-2 signaling are *Id1 *and *Smad6 *that encode products promoting the growth regulation of BMPs. The signaling pathway induced by BMP-2 can be modulated by numerous antagonist proteins, such as Noggin, Cerbarus and Gremlin. These antagonists are secreted in the extracellular matrix. Previous results using Noggin [26] and Chordin [31] support the potential therapeutic role of these antagonists in ovarian cancer progression through the inhibition of BMP signaling. It has also been reported that *Gremlin *gene expression is lower in ovarian cancer specimens compared to normal ovarian culture [28].

In the present study, we focused on the role of BMP-2 in ovarian cancer. First, we examined the biological role of BMP-2 on three novel ovarian cancer cell lines (TOV-2223, TOV-1946, TOV-112D). These lines were selected since they do not express detectable levels of BMP-2, consequently, their sensitivity and response to recombinant BMP-2 protein was examined. The ability of BMP-2 to induce signaling pathways and expression of target genes was investigated. Functional assays were also performed to determine the *in vitro *behavior of these cell lines in response to BMP-2 treatment. Finally the association between BMP-2 and ovarian cancer patient survival was examined using ovarian cancer tissue array analysis.

## Methods

### Cell culture and reagents

The TOV-2223, TOV-1946 and TOV-112D cell lines, developed from long term passages of serous ovarian cancer samples as described previously [32,33], were grown at 37°C in 5% CO_2 _and in OSE consisting of 50:50 medium 199:105 supplemented with 5% fetal bovine serum (FBS) and 2 μg/ml Gentamicin. All reagents used for cell culture media were purchased from Wisent (Qc, Canada). Human recombinant BMP-2 (355-BM-010/CF) and mouse Noggin (#1967-NG-025/CF) were supplied by R&D system (Mineapolis, MN, USA). TNF-α was obtained from Roche Applied Science (Indianapolis, IN). BMP-2-pCMV6-XL4 was purchased from Origene (Rockville, MD) and cloned into pcDNA3.1 (Invitrogen Life Technologies, Carlsbad, CA) as a *NotI *fragment. The pcDNA3.1-BMP-2 gene was sequenced to confirm the correct insertion of BMP-2 cDNA in the pcDNA3.1 vector.

### Primary cultures, tumor samples and patient characteristics

Tumor samples were collected from surgeries performed at the Centre hospitalier de l'Université de Montréal (CHUM). An independent pathologist assigned histopathology and tumor grade according to International Federation of Gynecology and Obstetrics (FIGO) criteria. A gynecologic oncologist reviewed tumor stage and residual disease. Normal tissues were obtained from tumor-free participants that have undergone oophorectomy. Primary cell cultures from normal ovarian surface epithelia (NOSE) and EOC samples were established as described [34,35]. Cells in primary culture were maintained in OSE media supplemented with 10% (v/v) fetal bovine serum (FBS), 2.5 ug/mL amphotericin B and 50 μg/mL gentamicin [34]. The tumor samples used for the tissue array studies are presented in Table 1. Tissue selection criteria for this study was based on all histopathologies from chemotherapy-naïve patients having provided informed consent with all samples having been collected between 1993–2003. Clinical data were extracted from the Système d'Archivage des Données en Oncologie (SARDO) that includes entries on tumor grade and stage, treatment and clinical outcomes such as the progression-free interval as defined by RECIST criteria and survival. No correlation between age of embedded paraffin tissues and antibody staining intensity on the tissue array was identified.

**Table 1:** Composition of the ovarian cancer tissue array

**Histopathology**	**Number of samples**	**Low stage**	**High stage**	**Grade 1**	**Grade 2**	**Grade 3**	**LMP**
Serous	19	3	17	6	4	7	2

Endometrioid	25	18	6	9	8	4	1

Clear cell	15	10	5	2	2	8	3

Mucinous	25	20	2	1	0	2	19

Mixed cells	5	2	3	0	0	4	1

### ELISA

Culture supernatants from confluent cellular monolayers were centrifuged at 3000 rpm for 10 min and frozen at -80C until further use. All ascites fluids were re-centrifuged for 10 min at 8000 rpm before performing ELISAs. After centrifugation, samples were tested by ELISA for secreted mature BMP-2 (item DBP200, R&D System) concentration according to the manufacturer's instructions. The limit of detection for BMP-2 was 30 pg/ml.

### RNA preparation and Quantitative PCR

Total RNA from cell lines was prepared using the RNeasy kit from Qiagen (Qiagen Inc., ON, Canada). The cDNA synthesis was done according to the protocol of the SuperScript™ First-Strand Synthesis System for real time PCR (Invitrogen Life Technologies, Carlsbad, CA) with a starting amount of 2 μg RNA and reverse transcription performed with random hexamers. The PCR reaction was performed with a Rotor-gene 3000 Real-Time Centrifugal DNA Amplification System (Corbett tumor tissues Research, NSW, Australia). The Quantitect™ SYBR Green PCR (Qiagen) reaction mixture was used according to the manufacturer's instructions. Serial dilutions were performed to generate a standard curve for each gene tested in order to define the efficiency of the real time PCR reaction and a melt curve was done to confirm the specificity of the reaction. Based on the strong stability of ERK1 gene expression in ovarian cancer tissue, it was chosen as an internal control [1]. All experiments, including positive and negative controls, were performed in triplicate. The PCR primers targeted exonic sequences that were interrupted by at least one intron. The amplicons were sequenced to verify their specificity for the targeted genes. Primers were: Id1 fw 5'-cggaatctgagggagaacaag, rev 5'-ctgagaagcaccaaacgtga; Smad6 fw 5'-gagctgagccgagagaaaga, rev 5'-agatgcacttggagcgagtt: Snail fw 5'-gagtggttcttctgcgctac, rev 5'-cagagtcccagatgagcatt; Wnt5a fw 5'-gcgcgaagacaggcatcaaag, rev 3'-ggcgttcaccacccctgctg; Erk1 fw 5'-gcgctggctcacccctacct, rev 5'-gccccagggtgcagagatgtc, BMPR1a fw 5'-cttattcagctgcctgtggt, rev 5'-attcttccacgatccctcct; BMPR1b fw 5'-tacaagcctgccataagtgaagaagc, rev 5'-tcatcgtgaaacaatatccgtctg and BMPR2: fw 5'-gctaaaatttggcagcaagc, rev 5'-cttgggccctatgtgtcact.

### Western blot analysis

Cells were lysed with cold lysis buffer (10 mM Tris-HCl, pH 7.4, 150 mM NaCl, 1 mM EDTA, 1 mM DTT/1 mM NaF/0.5% NP-40/0.5 mM PMSF/0.2 mM sodium orthovanadate/2 μg/ml of aprotinin, leupeptin and pepstatin), and the lysate boiled in loading buffer, separated by SDS-PAGE, and transferred onto a nitrocellulose membrane. Membranes were saturated with 5% (w/v) milk/PBS/0.1% Tween 20. Immunodetection was done as described in the ECL kit protocol (Amersham Pharmacia): i.e. incubated 2 h at room temperature with specific antibody, washed with PBS and incubated for another 30 min at room temperature with peroxidase-conjugated antibodies (Santa-Cruz Biotechnology Inc.). Western-blot analysis was performed with Erk1 (Santa Cruz Biotechnology Inc, CA), Smad1/5/8 (A-14 Santa Cruz Biotechnology Inc.), phospho-Erks (Cell Signaling, Beverly, MA, USA), phospho-Smad1/5/8 (Cell Signaling), p65 (Santa Cruz Biotechnology Inc), Akt (Santa-Cruz Biotechnology Inc.), phospho-Akt and phospho-S-536-p65 (Cell Signaling) and beta-Actin (AbCam, MA, USA) antibodies. All experiments were performed in triplicate with the TOV-2223 cell line and at least twice for the TOV-1946 and TOV-112D cell lines.

### Cytoplasmic and nuclear extracts

To prepare cellular extracts, 5 × 10^6 ^cells were washed twice in cold PBS buffer and resuspended in lysis buffer containing 10 mM Tris pH 7.9/10 mM NaCl/5 mM MgCl_2_/10 mM sodium orthovanadate/0.5 mM PMSF/10 μg/ml of the protease inhibitors (PMSF, pepstatin, leupeptin and aprotinin). After swelling the cells for 30 min on ice, 0.1% Nonidet P-40 and 10% glycerol (v/v) were added and the lysates centrifuged for 1 min at 4°C and 5,000 rpm. Supernatants consisting of cytoplasmic extracts were carefully decanted for cytoplasmic extracts. Nuclei pellets were resuspended for 1 h in 40 μl of lysis buffer containing 10 mM Tris pH 7.9/400 mM NaCl/0.1 EDTA/0.5 mM DTT/5% glycerol/0.5 mM PMSF/10 μg/ml protease inhibitors. Particulate matter was eliminated by centrifugation for 10 min at 13,000 × g at 4°C. Protein concentrations were determined using the Bradford method.

### Transfection and luciferase reporter assay

Cells were plated in 96-well plates and at 70–80% confluence (approximately 5 × 10^4 ^cells), they were co-transfected with 0.2 μg of DNA and 200 ng of a constitutively active Renilla luciferase (pCMV-RL) (Promega, WI, USA) by the lipofectamine method (Invitrogen Life Technology). After 6 h, cells were washed in fresh medium and incubated overnight. Cells were stimulated for 16 h with BMP-2 or TNFα and were then assayed for luciferase activity using the dual luciferase reporter assay system (Promega). The 3enh-κb-CONA-luc carries a firefly luciferase gene under the control of a trimeric repeat of the κB consensus [36].

### Cell proliferation

To determine the effect of BMP-2 on cell growth, 5 × 10^4 ^cells were plated into six well culture plates. After allowing the cells to adhere overnight they were treated with recombinant BMP-2 and/or Noggin. Two, four and six days later, cells were detached with trypsin and counted in the presence of 0.05% Trypan blue using a hemacytometer. Untreated cells were used as controls.

### Migration assays

Cells were grown to confluence in 6 well culture plates. Using a pipet tip, a wound was produced in the monolayer at two different positions on the plate. The adherent monolayer was then washed two times in PBS to remove non-adherent cells and media/FBS was added with or without BMP-2. After 20 or 40 hrs the open wound surface area was quantified by digital images taken under phase contrast microscopy. All experiments were repeated at least twice.

### Spheroid formation

Spheroids were formed using a modification of the hanging droplet method [37]. Briefly, 4 × 10^3 ^cells were resuspended in 16 μl of OSE/FBS media supplemented with 50 ng/ml BMP-2 and placed on the cover of a 150 mm tissue culture plate. The cover was placed over a plate that contained 15 ml of OSE to prevent dehydration of the hanging droplets. Spheroid formation was monitored after four and ten days, and representative spheroids were photographed. Untreated cells were used as controls.

### Tissue array and immunohistochemistry (IHC)

A tissue array containing 94 cores of ovarian epithelial tissues was built (Table 1, [1]). A detailed protocol is described in Le Page et al, [1]. Briefly, the tissue array was heated at 60°C for 30 min, de-paraffinized in toluene and rehydrated in a gradient of ethanol. Antigen retrieval was done in 90°C citrate buffer (0.01 M citric acid + 500 ul Tween-20/L adjusted to pH 6.0) (J.T. Baker Philipsburg, NJ) for 15 min. The tissue was blocked with a serum-free reagent (DakoCytomation Inc., Mississauga, ON) and incubated with BMP-2 antibodies (Santa-Cruz Biotechnology, CA, USA) overnight at 4°C in a humid chamber. Optimal antibody concentration was determined by serial dilutions. Endogenous peroxidase activity was quenched by treatment with 3% H_2_O_2_. The array was incubated with a secondary biotinylated antibody (DakoCytomation Inc.) followed by incubation with a streptavidin-peroxidase complex (DakoCytomation Inc.) for 10 min at room temperature. Reaction products were developed using diaminobenzidine containing 0.3% H_2_O_2 _as a substrate for peroxidase and nuclei were counterstained with diluted hematoxylin. Epithelial zones were scored according to the intensity of staining (value of 0 for absence, 1 for weak, 2 for moderate, 3 for high and 4 for very high intensity). Each array was independently analyzed in a blind study by two independent observers.

### Statistical analysis

For survival and progression-free disease analyses, we used the Cox regression survival model with time dependent covariate and Kaplan-Meier curves coupled with the log rank test. Receiver operating characteristics (ROC) curves were generated for each marker to define a threshold of expression corresponding to the best sensitivity and specificity for patient survival. A threshold of BMP-2 intensity = 4 appeared optimal. For Cox regression analysis, the markers were treated as categorical variables based on the threshold of expression. All statistical analyses were performed using SPSS software, version 11.0 (SPSS Inc., Chicago, IL, USA).

## Results

### Expression of BMP-2 in epithelial ovarian cancer cells

**Figure 1:**
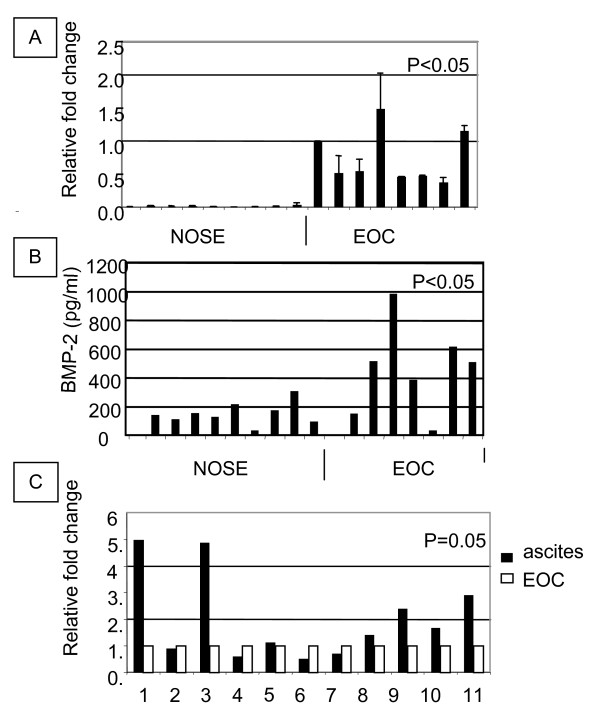
**A. BMP-2 expression in normal and malignant primary cultures**. RNA expression levels were normalized to that of the control RNA by real time RT-PCR assays. Relative fold change expression was the ratio of the first EOC sample to that of other samples. Values represent the mean +/- SEM of two experiments. **B. BMP-2 secretion in culture media of NOSE or EOC cell primary cultures**. Cell-free supernatants were collected and tested for BMP-2 concentration by ELISA. Values represent the mean of duplicate experiments. Significance as compared to NOSE samples was defined as p < 0.05 using a Student t-test. **C. BMP-2 mRNA expression in primary cultures of EOC from solid tumor and ascites**. Expression levels were quantified by real time RT-PCR and compared to the control RNA (Erk-1) Relative fold change expression was the ratio of the EOC from primary tumor sample to that of ascitic sample from the same patient. Values represent the mean of two experiments.

We have previously observed that BMP-2 expression is up-regulated in primary cultures of epithelial ovarian cancer cells and epithelial ovarian cancer tissues as compared to normal surface epithelial cells (Figure 1A and [1]). In addition, supernatants of primary cultures of cancerous cells showed higher concentrations of BMP-2 than supernatants from NOSE cells (Figure 1B) demonstrating that an active mature form of BMP-2 is expressed by ovarian cancer cells and could be release in the microenvironment. To further investigate the expression of BMP-2, we also compared its mRNA expression in matched malignant ascites cells and solid tumor from the same patient. As shown in Figure 1C, more than half of the patients tested showed higher expression of BMP-2 in tumor cells from ascites compared to tumor cells from solid tumors. This difference was statistically significant (p = 0.05, t-test).

### BMP-2 activates SMAD 1/5/8 and Erk MAPKs in ovarian cancer cell lines

To investigate the role of BMP-2 in ovarian cancer cells we selected three cell lines, TOV-2223, TOV-1946 and TOV-112D, for *in vitro *assays [33]. Microarray and RT-PCR analyses revealed that, although the three cell lines did not constitutively express BMP-2, they did express BMPR1a, BMPR1b and BMPR2 receptors (Table 2). Based on RNA expression, TOV1946 cells appear to express less BMPR2 receptor. In contrast the OV90 cell line [32] constitutively expressed BMP-2 but showed a very low expression of the corresponding receptors. These results suggested that the three cell lines, TOV-2223, TOV-1946 and TOV-112D could respond to endogenous stimulation with BMP-2.

**Table 2:** Gene expression of BMP receptors in ovarian cancer cell lines.

**Gene/Cell line**	**TOV-112D**	**TOV-1946**	**TOV-2223**
**BMPR1a**	6.40	0.28	3.46

**BMPR2**	25.10	0.11	1.0

**BMPR1b**	3.81	3.14	92.4

RNA was extracted, retro-transcribed and used for real-time PCR using specific primers for BMPR1a; BMPR1b; BMPR2

**Figure 2:**
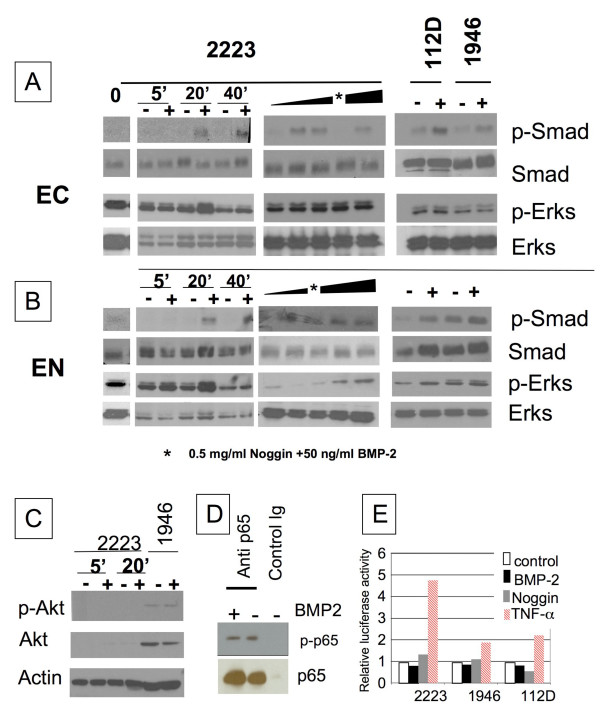
**Modulation of Smad, ERK, Akt and NF-κB activation by BMP-2**. **A and B**. TOV-2223 cells were stimulated in complete media for indicated times with 50 ng/ml BMP-2 (left) or for 20 min with increasing doses of BMP-2 (10, 50 or 100 ng/ml). TOV-112D and TOV-1946 cells were stimulated for 20 min with 50 ng/ml BMP-2. Cytoplasmic (EC) or nuclear (EN) extracts were subjected to western-blotting using anti-phosphoserine Smad1/5/8 and anti-phospho-tyrosine Erk1/2 antibodies. *Cells were stimulated with 50 ng/ml BMP-2 and 0.5 ng/ml Noggin **C**. Total extracts were subjected to western-blotting using anti-phosphoserine 473 Akt. TOV-2223 cells were stimulated for 5 or 20 min with 50 ng/ml BMP-2. TOV-1946 cells were stimulated with 50 ng/ml BMP-2 for 20 min. **D**. Total extracts from TOV-2223 cells were immunoprecipitated with anti-p65 antibody and loaded on an 8% polyacrylamide gel. Western-blotting was performed with anti-phosphoserine 536 p65. **E**. Cells were cotransfected with Renilla and 3κB-conA-luc vectors. Eight hours after transfection, cells were incubated with fresh media in the presence or absence of 50 ng/ml BMP-2 with or without 0.5 ng/ml Noggin or 10 ng/ml TNFα for 24 hrs. Cells were assayed for luciferase activity. Relative firefly luciferase activity was the ratio of luciferase activity in treated cells to that of non-treated cells. All experiments were repeated three times with similar results.

To determine whether the BMP-2 signaling pathways were functional in these cell lines, we examined the effect of BMP-2 treatment on three main signaling pathways. We first stimulated cells with BMP-2 and examined the phosphorylation and nuclear translocation of Smad1/5/8, since BMP-2 is thought to predominantly act through the activation of these transcription factors. The ability of BMP-2 to phosphorylate Smad1/5/8 was examined by western-blot using an antibody, which specifically recognizes the phosphorylated forms. As shown in Figure 2A, phosphorylation of Smad1/5/8 was induced after 20 minutes of treatment with as low as 10 ng/ml BMP-2 (Figure 2A). As expected the nuclear translocation of p-Smad1/5/8 was concomitant to their phosphorylation within the cytoplasm (Figure 2B). This effect was inhibited in presence of Noggin. Based on findings with TOV2223, the response to BMP-2 in TOV-112D and TOV-1946 cell lines was tested with the maximal dose of 50 ng/ml BMP-2 (Figure 2).

BMP-2 has also been shown to induce mitogenic signaling through the activation of Erk MAPKs [38]. In our ovarian cancer cell lines, constitutive phosphorylation of Erk MAPKs was visible and a slight transient increase was induced in the cytoplasm after 20 min of BMP-2 treatment (Figure 2A). This effect was more evident in the nuclear compartment and was dose-dependent as well as inhibited by the presence of Noggin (Figure 2B). Similar findings were seen in TOV-112D and TOV-1946 cell lines treated with 50 ng/ml BMP-2 (Figure 2).

Altogether, these results show that BMP-2 can induce a classical SMAD signaling pathway and that BMP receptors are functional in the three cell lines used. Consequently, these cell lines appear to be a good model to study BMP-2 effects on ovarian cancer cells.

### BMP-2 does not activate the Akt/NF-kB pathway

BMP-2 was also suspected to induce NF-kappaB (NF-κB) activation through the TAK1 and Akt pathways [29]. To determine if BMP-2 stimulation leads to NF-κB activation through the Akt pathway in ovarian cancer cell lines, we examined the phosphorylation of Akt and p65 using a specific antibody that recognizes phosphorylated serine sites. Neither constitutive nor BMP-2 induced phosphorylation of Akt was observed in TOV-2223 cells (Figure 2C) despite varying experimental conditions and film exposures. A weak and constitutive phosphorylation of Akt was seen in TOV-112D (not shown) and TOV-1946 (Figure 2C) cells but this basal level did not increase following BMP-2 treatment. Similarly, no constitutive or BMP-2 induced p65 phosphorylation was observed in TOV-2223 (not shown). While a weak constitutive phosphorylation of p65 was observed in TOV-112D cells, no increased phosphorylation was detected after 20 min of BMP-2 treatment (Figure 2D). To further confirm the absence of NF-κB activation by BMP-2, we examined the transcriptional activity of NF-κB following BMP-2 treatment. TNFα stimulation was used as a positive control. The transcriptional activity of NF-κB was measured by transient transfection of a reporter plasmid carrying a κB dependent promoter linked to a luciferase gene. After performing luciferase assays, no increases in κB dependent luciferase activity was observed in any of the cell lines tested (Figure 2E).

### BMP-2 increases *Id1*, *Smad6 *and *Snail *expression in ovarian cancer cell lines

**Figure 3:**
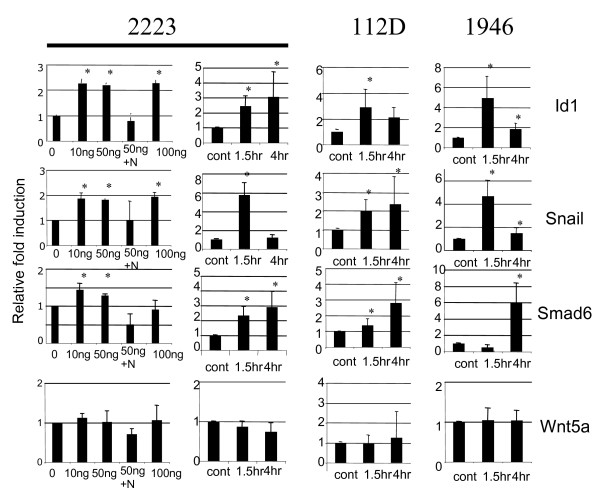
**Gene expression induced by BMP-2**. Cells were treated with 50 ng/ml BMP-2 in complete media or with indicated doses for 90 min. Where indicated, 0.5 ng/ml Noggin (N) was used. RNA was extracted, retro-transcribed and used for real-time PCR using specific primers for Id1, Smad6, Snail or Wnt5a. Relative fold change expression was the ratio of treated cells to that of non-treated cells. Values are the mean +/- SEM of duplicate wells from at least two independent experiments. * p < 0.05 (Student t-test).

We next examined whether the signaling pathways activated by BMP-2 led to the expression of known target genes of Smad such as ID1 and SMAD6. Since BMP-4 shares the same receptor with BMP-2, BMP4 may show effects similar to those seen with BMP-2, and therefore we also examined *Snail *expression, a BMP-4 regulated gene in ovarian cancer cells [26]. Real time RT-PCR analyses of each target gene showed that BMP-2 treatment increased *Id*1, S*nail *and S*mad6 *mRNA expression approximately two-fold (Figure 3). Time course experiments revealed that SNAIL expression was more transient than the induction of ID1 or SMAD6 expression.

A further increase in mRNA expression was not observed with doses higher than 10 ng/ml BMP-2. BMP-2 increased *Id1*, *Snail *and *Smad6 *expression in assays with TOV-223 and TO1946 cell lines but with slightly different kinetics than those seen with the TOV-2223 cell line. The largest fold change was observed in assays with TOV-1946 cells, which expressed the lowest constitutive level of *Id1*, *Snail *and *Smail6 *mRNA (data not shown). Wnt5a gene expression was used as negative control for gene expression assay as Wnt5a is not known to be regulated by BMPs. Indeed, q-RT-PCR showed that BMP-2 did not affect the expression of this gene confirming that the effect of BMP-2 on S*nail*, S*mad*6 and *Id1 *expression is specific to BMP-2 stimulation.

### BMP-2 affects the proliferation of ovarian cancer cell lines

**Figure 4:**
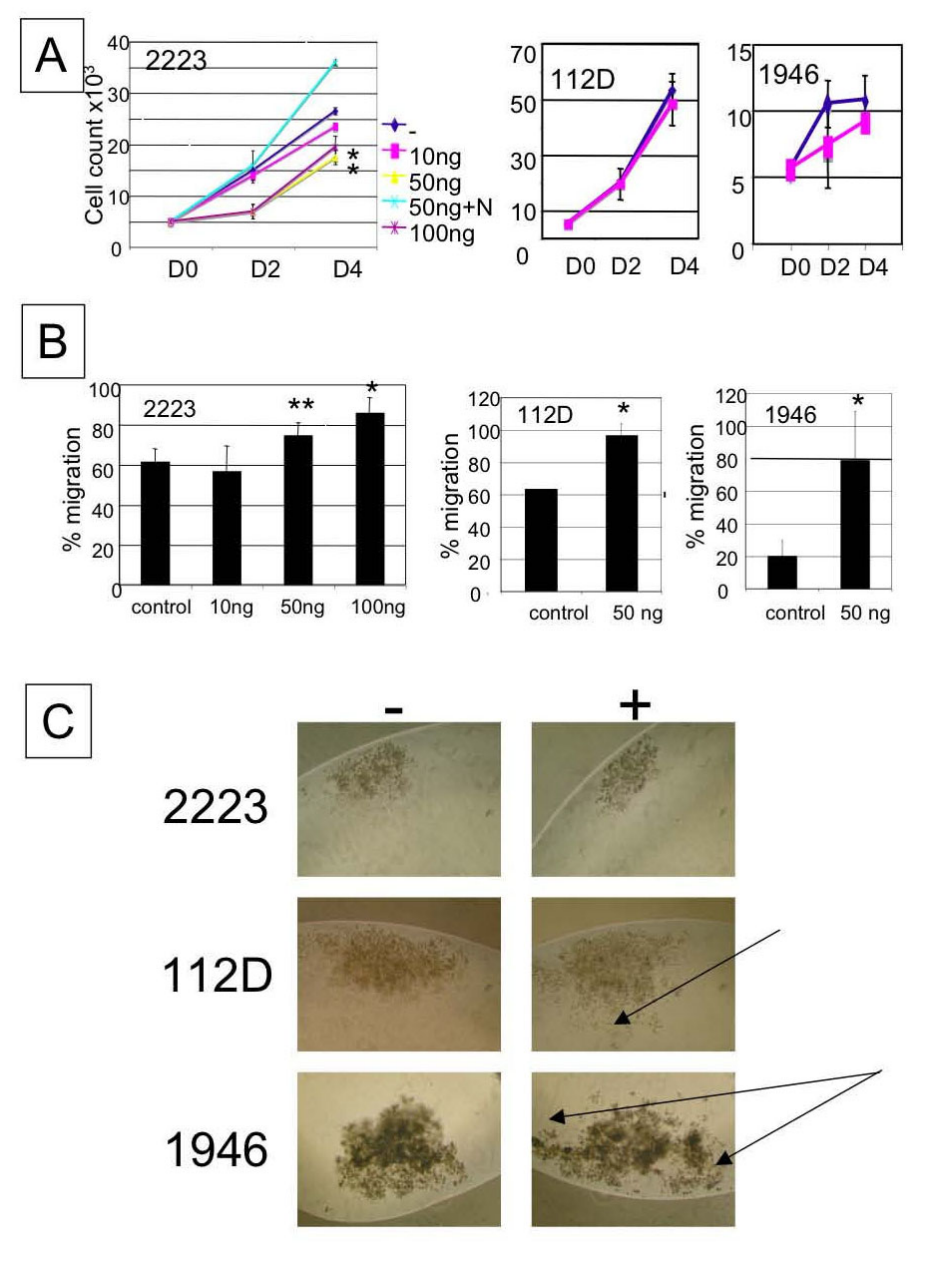
**Effects of BMP-2 on proliferation, migration and spheroid formation**. **A**. Cells were treated in complete media with indicated doses as indicated or with 50 ng/ml BMP-2 or left untreated for four days. Cells were counted every two days and media was changed every second day. Values are the mean +/- SEM of duplicate wells from at least two independent experiments. **B**. BMP-2 increases the motility of cells. Wounds were made on confluent monolayers of cells and then treated at indicated doses or with 50 ng/ml BMP-2. After 20 hrs (TOV-2223) or 40 hrs (TOV-112D and TOV-1946) the open wound surface area was quantified by digital images taken under a phase contrast microscope. Values are the mean +/- SEM of duplicates in at least two experiments and represent the percentage of total area covered by the cells in each image. **C**. Effect of BMP-2 on spheroid formation. Cells were cultured using a modification of the hanging droplet method. Cells were incubated in media with or without 50 ng/ml BMP-2. Spheroid formation was monitored after ten days. Arrows show scattered cells. All pictures were taken at a magnification of 100×. For all figures * p < 0.05, ** p < 0.10. (Student t-test).

We examined whether BMP-2 affects cellular proliferation of ovarian cancer cells. As seen in Figure 4A, BMP-2 decreased the cellular proliferation rate of TOV-2223 cells in a dose dependent manner. This effect was inhibited by the presence of Noggin. However the growth of either TOV-112D or TOV-1946 cell lines was not significantly affected by doses of 50 ng/ml BMP-2 or higher (Figure 4A) (data not shown).

### BMP-2 increases cellular migration of ovarian cancer cell lines

Cell migration in response to BMP-2 was estimated by the wound assays after 20 or 40 hours of treatment. The presence of BMP-2 in the culture media of TOV-2223 cells increased their motility in a dose dependent manner with a maximum effect being observed after 40 hrs of treatment with 100 ng/ml BMP-2 (Figure 4B). In TOV-112D and TOV-1946, a similar effect was observed after only 20 hrs of treatment with 50 ng/ml BMP-2 (Figure 4B).

### Effect of BMP-2 on spheroid formation

We have previously demonstrated that the TOV-112D and TOV-1946 cells are able to grow as spheroids as opposed to TOV-2223 [33]. Since the relationship between these three-dimensional structures and migration remains poorly defined, we determined the effect of BMP-2 on the formation of *in vitro *spheroids. For this purpose the cell lines were incubated either in the absence or presence of 50 ng/ml BMP-2. The formation of spheroids was determined after four and ten days. As expected, the TOV-2223 cells were not able to form compact spheroid and the presence of BMP-2 did not affect the spheroid formation (Figure 4C). In contrast, we noted significantly more cell scattering in the TOV-112D and TOV-1946 spheroids after treatment with BMP-2 (Figure 4C).

### BMP-2 is associated with a poor prognosis in ovarian cancer patients

**Figure 5:**
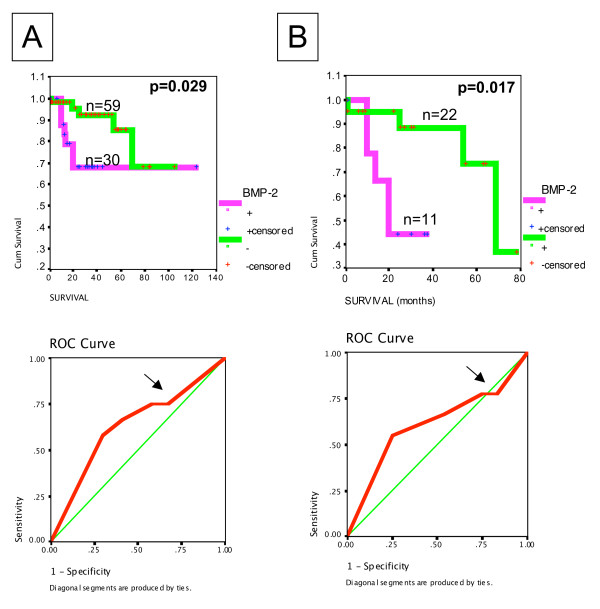
**Association between BMP-2 and survival**. Kaplan-Meier (top) and ROC (bottom) graphical representation of survival curves demonstrated a poorer survival associated with high expression of BMP-2 either in all tumors (A) or high stage (III–IV) tumors (B). Log-rank test was used to verify the significance of the difference in survival (p < 0.05).

We analyzed the association between patient outcome and BMP-2 protein expression by immunohistochemistry (IHC) using a tissue microarray of clinical samples from 89 ovarian cancer patients (Table 1 and Table 3) that was previously used to determine the potential of BMP-2 as a tumor marker [1]. Analysis of this tissue array showed that BMP-2 expression did not significantly correlate with age, stage or residual disease of patients. However, BMP-2 staining positively correlated with tumor grade (r = 0.25, p = 0.02, Spearman test). Kaplan Meier and Cox regression analyses also showed that BMP-2 expression was significantly associated with shorter survival period (p = 0.029 log rank) (Figure 5A) and with a high hazard ratio (HR = 3.475, 1.054–11.453). Since clinically low stage (I–II) disease has better outcomes that later stage (III–IV) disease, we also re-analyzed the data based on this stratification. Within low stage patients, BMP-2 was not associated with survival (p = 0.342, log rank) in contrast to high stage patients where there was significant association (p = 0.037, HR = 5.851, 1.112–30.787) (Figure 5B).

**Table 3:** Immunohistochemical staining of the ovarian cancer tissue array with an anti-BMP-2 antibody

**Staining:**	**0(n)**	**1(n)**	**2(n)**	**3(n)**	**4(n)**
					
**Histopatho:**					
**Serous**	4	2	3	5	5

**Endometrioid**	8	1	3	4	9

**Clear cell**	0	0	1	0	14

**Mucinous**	14	4	7	0	0

**Mixed cells**	2	0	0	1	2

N indicates the number of sample per staining intensity. Staining intensity is scored as 0: absent; 1: weak; 2:moderate; 3:strong; 4: very strong. (See also reference 1).

## Discussion

In this study we attempted to clarify the role of BMP-2 in ovarian cancer. An initial report highlighted the overexpression of BMP-2 in primary cultures of ovarian cancer cells and in the tissues of ovarian cancer patients [1]. Using three different cell lines, we report different *in vitro *and *in vivo *effects of BMP-2 on epithelial ovarian cancer cells. The three cell lines selected for this study expressed receptors for BMP-2 and were responsive to BMP-2 stimulation as seen by the activation and phosphorylation of Smad1/5/8 transcription factors as well as the gene expression of *Id*1, *Snail *and *Smad6*. However, although the signaling pattern was similar in all cell lines, they did not show the same biological activities in the presence of BMP-2. Only the TOV-2223 cell line showed a reduce proliferation rate in the presence of BMP-2 and was not influenced when cultured in 3D spheroid conditions. In contrast, the motility of all cell lines was stimulated in presence of BMP-2. Further work needs to be done to define particular characteristic of each ovarian cancer cell line that determine response to BMP-2. These results suggest that the effects of BMP-2 on ovarian cancer cells may be complex and dependent on the particular cellular context. The heterogeneity in response to BMP-2 is unlikely related to the histopathological subtype since TOV-2223 and TOV-1946, which respond differently to the presence of BMP-2, are both derived from a serous subtype.

Similar effects with BMP-4, as observed here with BMP-2, have recently been reported in ovarian cancer cell lines and ovarian cancer primary cultures [26,39]. We observed that BMP-2 slightly reduced the proliferation of TOV-2223 cells but had no effect on TOV-112D and TOV-1946 cells suggesting that some cell lines are resistant to the anti-proliferative activity of BMP-2. In the same way, BMP-4 has also been reported to slightly reduce the proliferation of SKOV3 ovarian cancer cells, as well as some primary cultures of ovarian cancer cells while other ovarian primary cultures were not sensitive to this protein [39]. The reason why some ovarian cancer cells are resistant to this anti-proliferative effect is unknown. We also observed similar increases in motility in cells treated with BMP-2 as reported by others with BMP-4 [26]. Since BMP-2 and BMP-4 bind the same type I and type II BMP receptors, it is not surprising to notice similarities in their induction of signaling pathways. A strategy based on the single inhibition of either BMP-2 or BMP-4 may not be sufficient to reduce the tumorigenic effect driven by Smad1/5/8 signaling. In contrast, targeting several BMPs by the use of extracellular antagonists such as Chordin, Noggin or Gremlin may be more effective. Preliminary results shown here with Noggin and by others using Noggin [26] and Chordin [31] support the potential therapeutic role of these antagonists in ovarian cancer progression through the inhibition of BMP signaling. It will be of great interest to test Noggin, Chordin, Cerberus or Gremlin as *in vivo *potential tumor suppressors in xenograph models.

We also observed that some malignant cells from ascites samples overexpressed BMP-2 compared to cells from solid tumor samples of the same patients. The motility of cancer cells is an important factor determining the metastatic spread of tumors. As ascites tumor cells are detached from the primary tumor site and may have acquired a metastatic potential, this observation suggests that BMP-2 may be associated or involved in the process of evading tumor cells from the primary site to the omentum. In line with this hypothesis, we observed that BMP-2 stimulates the *in vitro *migration of ovarian cancer cell lines. In addition several reports have shown a role of BMP-2 in invasion of lung, prostate, breast cancer cells and BMP-4 in ovarian cancer [21,40,41]. To confirm the role of BMP-2 in the metastatic process of ovarian cancer cells, additional *in vivo *assays would be required. Metastasis is a major cause of cancer related mortality. The fact that patients with higher expression of BMP-2 in ovarian tissues have shorter survival supports a role for BMP-2 in the motility of ovarian cancer cells and aggressiveness of ovarian tumors. Further functional assays are required to determine the exact role of BMP-2 in these biological processes.

## Conclusion

In conclusion, the evidence provided in this study support the fact that BMP-2 overexpression may modulate cellular motility and cellular adherence. In addition, we show that high expression of BMP-2 in ovarian cancer tissues is associated with shorter survival in patients.

## Competing interests

The authors declare that they have no competing interests.

## Authors' contributions

Conception, coordination and design of the study: CLP, PT and AMMM. Financial support to: PT, DMP and AMMM. Collection and analysis of clinical data: CLP, MdeL and DMP. Collection and analysis of molecular data: CLP, MP, MZ and LM. Collection and Assembly of data: CLP.  Data analysis and interpretation: CLP, MdeL, MZ and LM. Manuscript writing: CLP and AMMM.
